# Clinical, genetic and quality-of-life study of a cohort of adult patients with tuberous sclerosis

**DOI:** 10.1186/s13023-021-01878-2

**Published:** 2021-05-31

**Authors:** Elena Cristina De Sautu De Borbón, Juan Manuel Guerra Vales, Carlos Lumbreras Bermejo, Felix Guerrero Ramos, María José Buj Padilla, Jesús González de la Aleja, Montserrat Morales Conejo

**Affiliations:** 1 Department of Internal Medicine, University Hospital, 12 de Octubre, Avenida de Córdoba s/n, 28041 Madrid, Spain; 2 Research Institute I+12, University Hospital, 12 de Octubre, Avenida de Córdoba s/n, 28041 Madrid, Spain; 3 Department of Urology, University Hospital, 12 de Octubre, Avenida de Córdoba s/n, 28041 Madrid, Spain; 4 Department of Radiology, University Hospital, 12 de Octubre, Avenida de Córdoba s/n, 28041 Madrid, Spain; 5 Department of Neurology, University Hospital, 12 de Octubre, Avenida de Córdoba s/n, 28041 Madrid, Spain

**Keywords:** Tuberous sclerosis, Adult, Quality of life, Multidisciplinary unit

## Abstract

**Background and objective:**

Tuberous sclerosis (TS) is a condition whose manifestations in childhood have been extensively described, but whose presentation in adults is less well known. This study describes the clinical and genetic characteristics, therapeutic management and quality of life of a cohort of adult patients with TS. A comparative study of the characteristics of patients diagnosed in childhood and adulthood is also carried out.

**Material and methods:**

This observational, retrospective, cross-sectional study included a large cohort of adult patients (≥ 16 years old) followed for 5 years in a specific rare diseases unit.

**Results:**

Fifty-seven patients with a diagnosis of tuberous sclerosis were included, more than 50% of whom were diagnosed as adults. The mean age of the patients was 42 years (20–86). The central nervous system was the main area affected (97%), followed by the skin (80.7%) and kidneys (73%). The most frequent genetic alteration was a mutation in the *TSC2* gene (47.7%). Among patients diagnosed in adulthood, there was less neurological involvement, with less frequency of epileptic seizures (30.8% vs 60.79% of patients diagnosed in childhood) and astrocytomas (3.8% vs 53.6%), less intellectual disability (11.5% vs 71.4%) and less expressiveness of the condition. 42% of patients were treated with mTOR pathway inhibitors, and presence of an angiomyolipoma was the main indication. In a quality-of-life analysis, the means of the summary indices were below the scores of the average Spanish population: (47.42 (SD ± 9.82) on the physical health scale, 45.61 (SD ± 7.99) on the mental health scale) versus 50 (SD ± 10) for the general population.

**Conclusions:**

Up to 50% of adult patients with TS were diagnosed in adulthood, and the condition is less severe with less frequent epileptic seizures and intellectual disability. 42% require treatment with mTOR inhibitors, in most cases due to the presence of AMLs. The quality of life of adult patients with TS is diminished compared to the general population.

## Introduction

Tuberous sclerosis (TS) or tuberous sclerosis complex (TSC) is an autosomal dominant neurocutaneous disorder with full penetrance and variable expressivity, characterized by the formation of benign tumors (hamartomas) in multiple organs (brain, skin, kidneys, retina, heart and lungs) [1]. The prevalence of TS ranges from 7 to 12 cases per 100,000 inhabitants and the incidence at birth is 1 case per 5000–10,000 newborns [2-5].

Mutations in one of two tumor suppressor genes (*TSC1* and *TSC2*) have so far been identified as the cause of TS. These genes encode two proteins (hamartin and tuberin) which form a complex, whose functions include cell growth inhibition by inhibiting mammalian target of rapamycin complex 1 (mTORC1) [6, 7].

Diagnosis is based on genetic and/or clinical criteria established at the International Tuberous Sclerosis Complex Consensus Conference in 2012 [7]. Patients require close monitoring to assess disease progression and the appearance of new lesions.

mTOR inhibitors have been used in recent years for the treatment of this condition. The accepted indications for these drugs are the presence of growing asymptomatic subependymal giant cell astrocytomas (SEGA), renal angiomyolipoma with lesions larger than 3 cm in diameter and growing, lymphangioleiomyomatosis (LAM) with moderate, severe, or rapidly progressive involvement, and epilepsy refractory to treatment [8]. Trials conducted so far with mTOR inhibitors have shown good effectiveness and safety [9-16].

TS is a multisystem disorder in which phenotypic expression can vary over the years, with neurological and cutaneous manifestations being more prevalent in childhood, and kidney and pulmonary involvement more characteristic of adulthood. Because of this, it is necessary to ensure a structured follow-up throughout the patient’s life, which will necessarily involve different specialists. The creation of multidisciplinary units to tackle the condition enables comprehensive integrated patient management [17-19]. These teams should include both pediatricians and internal medicine specialists to facilitate the transition to adulthood.

TS is a condition that is usually reported in childhood, while less is known about it when it presents in adults [18, 20]. The objective of this study is to describe the clinical manifestations of a cohort of adult patients followed in a special multidisciplinary unit of a tertiary hospital, collecting clinical and genetic characteristics, details of therapeutic management and quality of life, and finally to carry out a comparative study of the characteristics of patients diagnosed in childhood and adulthood, as well as the evolution of patients treated with mTOR inhibitors.

## Material and methods

This retrospective, observational, cross-sectional study was conducted in the Unit for Rare Diseases in Adults at the University Hospital 12 de Octubre in Madrid, Spain, and included all patients aged ≥ 16 years with a diagnosis or high suspicion of TS who were being followed after a consultation at the unit between September 2013, when the unit opened, and January 2019. The diagnosis of TS was made in accordance with the recommendations of the International Tuberous Sclerosis Complex Consensus Conference of 2012 [7].

For collection of variables, an ad hoc program was designed using the Windows Excel program. Data were collected from the electronic clinical record. Patient follow-up was in accordance with standard clinical practice, with an annual patient assessment if the condition remained stable, and every 4–6 months if receiving mTOR inhibitor treatment or if monitoring was necessary for progression of some clinical sign or symptom.

All patients were assessed on every occasion by an Internal Medicine specialist with expertise in rare diseases, and by other specialists depending on the needs of the patient.

A descriptive analysis of the variables collected was carried out. Continuous variables were presented as means (with standard deviation) for normally distributed data, and as medians when they did not follow a normal distribution; categorical variables were presented as frequencies (n) or percentages (%). 2 × 2 or n × 2 contingency tables were constructed to study frequency distributions.

Qualitative variables were compared using the chi-squared test, or the Fisher’s exact test. Inferences for continuous variables were estimated after first applying the Student’s t-test.

The SF-36 questionnaire was used to evaluate quality of life. The questionnaire manual (Spanish Version of SF-36v2™ Health Survey© 1996, 2000 adapted by J. Alonso et al., 2003) was used to score the eight scales of the questionnaire and calculate the two physical and mental summary measures. Missing values for any of the items were imputed, provided that 50% of all items on the scale in question had been answered. Pearson’s correlation coefficient was used to study correlations between the eight scales of the questionnaire, and the two summary measures. The Student’s t-test (two groups) or ANOVA (more than two groups) were used to compare scores between different groups.

All analyses were carried out using SPSS version 21.0 (SPSS Inc. Chicago, IL, USA). Values of p < 0.05 were considered statistically significant.

## Results

### General aspects

A total of 61 patients were assessed up to January 2019, 4 of whom were excluded because they did not meet the definitive criteria. The final cohort studied comprised 57 patients (27 men (47.4%) and 30 women (52.6%)) with a mean age of 42 years (20–86). The demographic characteristics of the population studied are listed in Table 1.

**Table 1:** Demographic data

Demographic data (*N* = *57*)	
Age at diagnosis, n (%)	
Adult	26 (45.6%)
Childhood	28 (49.12%)
Sex, n (%)	
Woman	30 (52.6%)
Man	27 (47.4%)
Mean age	41.96 (SD ± 14.4)
Reason for referral	
Multidisciplinary consultation	40 (70.2%)
Affected family member	13 (22.8%)
Transition to adulthood	4 (7%)
Family affected	22 (38.59%)
Genetic study, n (%)	44 (77.2%)
No mutation	4 (9%)
*TSC1*	11 (25%)
*TSC2*	21 (47.8%)
Mutation of uncertain significance	8 (18.2%)

With respect to the clinical diagnostic criteria for tuberous sclerosis (Table 2), half of the patients (50.8%) satisfied between 3 and 5 of the major criteria, and 15.8% of the patients more than 7 criteria. With respect to the minor criteria, 52.6% had none, 35.1% had one, and 12.3% two minor criteria.

**Table 2:** Diagnostic criteria

Genetic	Identification of pathogenic mutations of *TSC1* or *TSC2* in DNA from healthy tissue is sufficient to establish a definitive diagnosis of tuberous sclerosis complex	
Clinical	Major	Minor
	Hypomelanotic macules (≥ 3, at least 5 mm in diameter)	Confetti-like skin lesions
	Angiofibromas (≥ 3) or fibrous cephalic plaque	Pitted teeth (> 3)
	Ungual fibromas (≥ 2)	Intraoral fibromas
	Shagreen patch	Retinal achromic patch
	Multiple retinal hamartomas	Multiple renal cysts
	Cortical dysplasia*	Extra-renal hamartomas
	Subependymal nodules	
	Subependymal giant cell astrocytoma	
	Cardiac rhabdomyomas	
	Lymphangioleiomyomatosis (LAM)**	
	Angiomyolipoma (≥ 2)**	
Definitive diagnosis:	2 major criteria or 1 major criterion + ≥ 2 minor criteria	
Possible diagnosis	1 major criterion or ≥ 2 minor criteria	

*Includes tubers and cerebral white-matter radial migration lines

**LAM and angiomyolipomas on their own without other alterations do not meet the criteria

### Description of the clinical characteristics of the adult patients with TS

The predominant clinical manifestations in our cohort are listed in Table 3. The main clinical involvement was neurological (97%), followed by dermatological (80.7%) and renal (73.7%).

**Table 3:** Clinical manifestations

Clinical manifestations	
Neurological	
Cortical dysplasia	49 (86%)
Epileptic seizures	38 (66.6%)
Subependymal nodule	32 (56.1%)
SEGA	17 (29.8%)
Renal	
Renal angiomyolipoma	35 (61.4%)
Multiple renal cysts	25 (43.8%)
Pulmonary	
Lymphangioleiomyomatosis	16 (28.1%)
Cardiovascular	
Cardiac rhabdomyoma	16 (28.1%)
Cutaneous	
Facial angiofibroma	40 (70.2%)
Hypomelanotic macules	25 (43.9%)
Periungual fibromas	25 (43.9%)
Ophthalmological	
Retinal hamartoma	10 (17.5%)
Other	
Sclerotic bone lesions	26 (45.6%)
Liver angiomas	16 (28.1%)

#### Neurological manifestations

The most frequent neurological manifestations were cortical tubers and subependymal nodules, with a prevalence of 49/57 (86%) and 32/57 (56%) respectively. SEGA was diagnosed in 17/57 (29.8%) of patients. Of the 17 patients with SEGA, 8 had undergone surgery in childhood.

Cranial MRI was performed in 89.5% of the patients. In 49.1% (28 patients), the lesions remained stable in follow-up tests, and in the rest, there were no previous test results with which to compare their evolution.

Thirty-eight (66.6%) of the patients suffered epileptic seizures (Table 4). In twenty-six of these (68.4%), the episodes were controlled and there had been no new ones in the past year; in 5/38 (13.2%), the seizures were controlled but there had been some seizure in the past year, and in 7/38 (18.4%) of patients, the seizures were uncontrolled. Of the patients with epilepsy, 14/38 (36.8%) presented as generalized tonic–clonic seizures, 8/38 (21.1%) as absence seizures. The rest of the cases had different types of seizure, or it was not possible to obtain the information. The mean number of antiepileptic drugs prescribed to epilepsy patients was 1.82 (SD ± 1.27), with a range between 0 and 5.

**Table 4:** Characteristics of patients with epilepsy

Epileptic seizures	N = 38
Age of onset	
Childhood	30 (78.9%)
Adult	3 (7.9%)
Not available	5 (13.2%)
Control of seizures	
Controlled with no seizure(s) in past year	26 (68.4%)
Controlled with seizure(s) in past year	5 (13.2%)
Uncontrolled	7 (18.4%)
AEDs (mean number of drugs used)	1.82 (SD ± 1.27)
Other treatments	
Vagus nerve stimulation	2 (5.3%)
Epilepsy surgery	2 (5.3%)
Everolimus	3 (7.8%)

With regard to cognitive alterations, 31 patients (54.4%) had no intellectual disability, 14/57 (24.6%) had mild disability, 5/57 (8.8%) moderate, and 7/57 (12.3%) of the patients were classified as severely disabled. Patients with the worst control of epileptic seizures were also observed to be those with the greatest intellectual disability. Of the patients without disability, 17/31 (54.8%) were seizure free and only 1/31 (3.2%) had uncontrolled seizures, which contrasts with the severely disabled patients, 4/7 (57.1%) of whom had uncontrolled seizures and none was seizure free. These differences were statistically significant *p* < 0.0001.

#### Renal manifestations

Cysts were found in 25/57 (43.8%) of patients and angiomyolipomas in 35/57 (61.4%). Some 14/57 (24.5%) of patients had arterial hypertension and 7/57 (12.3%) showed deteriorating kidney function. Among the patients with renal AMLs, 2 patients (5.7%) had a single AML, 5 patients (14.3%) had several (2–3) and 28 patients (80%) had multiple AMLs (> 3). In four patients, the size of the AML was between 3 and 5 cm, in another four, between 5–8 cm, in thirteen, more than 8 cm, and in 14 patients < 3 cm. Bilateral renal AMLs were found in 94.3% of patients. The most frequent clinical manifestations were deterioration of renal function in seven patients, spontaneous bleeding in two patients and hematuria in another patient.

Among the treatments used for patients with AML, mTOR inhibitors were the most frequent (in 57% of cases). After the histories and clinical data of the 35 patients with AMLs were reviewed, it was found that, prior to the start of follow-up in our unit and before use of mTOR inhibitors, 5 patients (14.3%) had undergone nephrectomies, six (17.1%) one or several embolizations, and 4 patients (11.4%) had required kidney transplants due to end-stage kidney disease.

#### Pulmonary manifestations

Lymphangioleiomyomatosis (LAM) was found in 16 patients (28.1%), all of them women (16/30, 53.3% of the women). Most patients were asymptomatic. The main clinical pulmonary manifestation was a pneumothorax in five patients (31.3%). Of the patients with LAM, ten (62.5%) were classified as mild, five (31.3%) as moderate, and one (6.3%) as severe.

#### Cutaneous manifestations

The most common cutaneous manifestation was facial angiofibromas (40/57, 70.2% of patients with TS). Also reported were hypomelanotic macules, in 25/57 (43.9%) of patients, fibrous cephalic plaques in 1/57 (1.8%), shagreen patches in 13/57 (22.8%), confetti skin lesions in 6/57 (10.5%), periungual fibromas in 25/57 (43.9%), intraoral fibromas in 5/57 (8.8%) and dental enamel lesions in 2/57 (3.5%).

#### Other manifestations

Cardiac involvement was detected in 16 patients (33.3% of those who had specific studies) in the form of cardiac rhabdomyomas, all of whom already presented residual tumors. No associated arrhythmias were reported. Retinal hamartomas were described in 10 (17.5%) of the patients, taking into account that 31/57 (54.4%) of the patients had never been assessed by an ophthalmologist. No cases of secondary visual alterations were found.

### Description of the genetic characteristics of adult patients with TS

Genetic screening, involving next-generation sequencing (NGS) panel and multiplex-ligation probe amplification (MLPA) analysis [21, 22], was conducted in 44/57 (77.2%) of the patients: eleven (25%) patients had a mutation in the *TSC1* gene, 21/44 (47.8%) in the *TSC2* gene, 8/44 presented mutations of uncertain significance, and in 4/44 patients (9%), no variants that could be considered potentially pathogenic were detected.

With respect to the clinical manifestations, patients with a proven genetic predisposition had greater renal involvement and more AMLs. However, no differences of neurological involvement were seen in the form of seizures, nor in terms of pulmonary, cutaneous, cardiac or ophthalmological involvement.

When the manifestations of patients were examined according to whether they had mutations in the *TSC1* or *TSC2* gene, the only statistically significant differences seen were seen in the appearance of more renal AMLs in patients with the *TSC2* mutation, but not in other types of involvement. At the same time, only patients with mutations in *TSC2* required treatment with mTOR inhibitors.

### Differences in clinical manifestations according to age at diagnosis

Twenty-eight patients (49.12%) were diagnosed in childhood (≤ 16 years old) and 26/57 (45.6%) in adulthood. Those who were diagnosed in childhood had more major diagnostic criteria (5.82 ± 1.517) than those who received their diagnosis in adulthood (3.08 ± 1.623) (*p* < 0.0001).

The clinical presentation at diagnosis was epilepsy in 23/28 (82.1%) of patients diagnosed in childhood. Of the patients diagnosed in adulthood, 13/26 (50%) were related to kidney and skin affectations, and the other 13/26 (50%) were diagnosed following screening for TS as a result of having an affected family member.

With regard to genetic predisposition, patients diagnosed in childhood more frequently presented mutations in the *TSC2* gene 12/28 (43%) versus 4/28 (14%) in *TSC1*. Among those diagnosed in adulthood, 7/26 (27%) had mutations in *TSC1*, 14/26 (53.8%) mutations in *TSC2*, with 6/14 (42.8%) of the latter mutations being of uncertain significance.

Patients diagnosed in childhood had greater neurological involvement at all levels: in the form of seizures, the presence of subependymal nodules and astrocytomas, although this difference was not found in cortical dysplasia. Patients diagnosed as children also has greater intellectual disability, which was practically non-existent in patients diagnosed as adults. Renal involvement was more frequent and severe in patients diagnosed in childhood, who also had more dermatological and cardiac involvement, although no differences were found at the pulmonary or ophthalmological level.

(Table 5).

**Table 5:** Clinical manifestations according to age at diagnosis

	Age at diagnosis		
	Childhood (N = 28)	Adult (N = 26)	*p*-value
Seizures			
No	17.9% (5)	53.8% (14)	**0.038**
Yes, controlled, no seizure in 1 year	60.7% (17)	30.8% (8)	
Yes, controlled, seizure in last year	10.7% (3)	3.8% (1)	
Uncontrolled	10.7% (3)	11.5% (3)	
TAND			
No	28.6% (8)	88.5% (23)	** < 0.0001**
Mild	39.3% (11)	7.7% (2)	
Moderate	17.9% (5)	0% (0)	
Severe	14.3% (4)	3.8% (1)	
Cortical dysplasia			
No	17.9% (5)	11.5% (3)	0.514
Yes	82.1% (23)	88.5% (23)	
Subependymal nodule			
No	25% (7)	65.4% (17)	**0.003**
Yes	75% (21)	34.6% (9)	
Astrocytomas			
No	46.4% (13)	96.2% (25)	** < 0.0001**
Yes	53.6% (15)	3.8% (1)	
AML			
No	10.7% (3)	69.2% (18)	** < 0.0001**
Single	0% (0)	7.7% (2)	
Various	10.7% (3)	7.7% (2)	
Multiple > 3	78.6% (22)	15.4% (4)	
LAM			
No	67.9% (19)	73.1% (19)	0.676
Mild	21.4% (6)	15.4% (4)	
Moderate	7.1% (2)	11.5% (3)	
Severe	3.6% (1)	0% (0)	
Skin			
No	7.1% (2)	30.8% (8)	**0.026**
Yes	92.9% (26)	69.2% (18)	
Rhabdomyoma			
No	57.1% (16)	84.6% (22)	**0.027**
Yes	42.8% (12)	15.4% (4)	
Retinal hamartoma			
No	71.4% (20)	92.3% (24)	0.048
Yes	28.6% (8)	7.7% (2)	
mTOR inhibitors			
No	32.1% (9)	84.6% (22)	**< 0.0001**
Yes	67.9% (19)	15.4% (4)	

Bold highlights statistically significant results

TAND, TSC-associated neuropsychiatric disorders; AML, angiomyolipoma; LAM, lymphangioleiomyomatosis; mTOR, Mammalian target of rapamycin (mTOR) inhibitors

*54 of 57 due to absence of information in 3 patients

The need for treatment with mTOR inhibitors was higher among patients diagnosed in childhood compared to those diagnosed as adults (67.9% vs 15.4%; *p* < 0.0001). Four out of 26 patients diagnosed in adulthood received treatment with an mTOR inhibitor, and in all 4 patients, the indication for treatment was the presence of renal AMLs.

### Description of patients receiving treatment with mTOR inhibitors

Treatment with mTOR inhibitors was prescribed in 24 patients (42.1%). Indications for starting treatment were epilepsy in 3 patients (5.3%), SEGA in one patient (1.8%) and renal AML in 20 patients (35.1%).

The patient being treated for SEGA remained stable throughout the 5 years of treatment. The brain lesion remained stable (without the need for surgery), as did the epileptic seizures and renal AMLs that he also presented.

Two of the 3 patients with an indication for epileptic seizures were controlled, and treatment was maintained for 6 and 8 years (they were included in the first clinical trials). In the third patient, treatment was suspended due to lack of effectiveness.

In 10 of the patients treated for renal AMLs, the lesions remained stable over time and in 4 patients, they decreased in size.

The mean treatment duration was 5 years; four patients were treated for more than 8 years.

The drug had to be suspended during treatment in 7 patients (29.1%) due to side effects, in 4 patients (16.6%) prior to surgery, and due to lack of effectiveness in one (4.16%) patient. It was later reintroduced in 9 of these patients and was subsequently well tolerated. Side effects were reported in 16 patients (66.6%), the most frequent being stomatitis (9 patients) which was controlled with medication in all but one case, which required a temporary suspension.

The dose also had to be lowered in 3 patients due to a dry cough at night with no evidence of pneumonitis, which improved the symptoms. 90% of the patients presented dyslipidemia, which made it necessary to use statins, and 3 presented mild hypophosphatemia that did not require treatment. Weight loss (about 5 kg) and prolonged periods without menstruation were common in women. Most showed evidence of improvement in cutaneous angiofibromas, and no major infections were described. All patients had previously been screened for TB, HBV, HCV and appropriate vaccinations. No other major drug-related side effects were described.

### Quality-of-life analysis

A quality-of-life assessment was conducted with 49 patients using the SF-36 questionnaire. Means and standard deviation were calculated for each of the SF-36 subscales and two summary measures (a physical health summary index (PSI) and a mental health summary index (MSI). Subscale values ranged from 0 to 100, with higher scores corresponding to a better state of health. Mean scores of more than 70 points were obtained for the physical functioning, physical role, body pain, social functioning and emotional role scales. The results were worse on the general health (the worst affected), vitality and mental health scales.

The summary indexes are standardized and provide a direct interpretation of the scores relative to the general Spanish population, which has a mean of 50 and SD ± 10.

The physical and mental summary indices were calculated, giving mean scores of 47.42 (SD ± 9.82) on the physical scale, and 45.61 (SD ± 7.99) on the mental scale, both below the population average.

The quality-of-life scores of the patients in our cohort were compared with quality-of-life results published for the general Spanish population. Quality of life in our cohort was observed to be significantly decreased relative to the general population on the scales for general health (56.97 vs 68.3 *p* = 0.0194), general vitality (58.36 vs 66.9 *p* = 0.0221), social functioning (80.35 vs 90.1 *p* = 0.0385) and mental health (65.46 vs 73.3 *p* = 0.0216). Considering that our sample has a mean age of 42 years, these values would be found in the 20th percentile of the general population.

The clinical manifestations with the greatest effect on quality of life were evaluated (Table 6). The neurocognitive commitment, and hence the presence of epileptic seizures, was found to impair quality of life most, particularly in the physical component, as well as the presence of astrocytoma.

**Table 6:** Quality of life

	Standardized physical component Mean (SD)	Standardized mental component Mean (SD)	*p*-value
Seizures			
No (N = 17)	50.11 (8.79)	47.59 (7.14)	PC:0.054
Yes, controlled, no seizures in 1 year (N = 23)	48.38 (8.29)	44.12 (8.16)	
Yes, controlled, seizures in last year (N = 4)	42.83 (10.16)	50.05 (7.91)	MC:0.264
Uncontrolled (N = 5)	37.52 (14.61)	42.15 (9.23)	
TAND			
No (N = 26)	49.39 (8.55)	47.21(6.47)	**PC: 0.002**
Mild (N = 13)	50.75 (6.44)	42.49 (10.31)	
Moderate (N = 5)	41.96 (8.64)	49.94 (4.56)	MC: 0.104
Severe (N = 5)	33.98 (13.33)	41.08 (8.17)	
Cortical dysplasia			
No (N = 6)	50.85 (6.92)	47.40 (6.21)	PC: 0.211
Yes (N = 43)	46.94 (10.13)	45.36(8.24)	MC: 0.378
No (N = 22)	46.86 (9.76)	44.09 (8.43)	PC: 0.994
Subependymal nodule			
Yes (N = 27)	47.88 (10.04)	46.85 (7.54)	MC: 0.628
Astrocytoma			
No (N = 34)	48.73 (8.04)	45.44 (8.71)	**PC: 0.008**
Yes (N = 15)	44.46 (12.84)	45.99 (6.32)	MC: 0.198
AML			
None (N = 16)	50.41(7.60)	47.37 (7.57)	PC: 0.461
Single (N = 2)	49.12 (7.72)	46.48 (5.41)	
Several (N = 5)	46.16 (4.33)	47.74 (6.66)	MC: 0.555
Multiple > 3 (N = 26)	47.42 (11.62)	44.05 (8.63)	
LAM			
No (N = 34)	47.74 (10.02)	45.58 (7.64)	PC: 0.851
Mild (N = 10)	48.08 (9.47)	47.09 (4.96)	
Moderate (N = 4)	44.77 (11.96)	46.59 (13.84)	MC: 0.145
Severe (N = 1)	40.76	27.81	
Skin			
No (N = 8)	52.61 (7.73)	48.62 (6.52)	PC: 0.297
Yes (N = 41)	46.41 (9.95)	45.02 (8.18)	MC: 0.536
Rhabdomyoma			
No (N = 33)	48.00 (9.90)	45.91 (7.53)	PC: 0.739
Yes ( N = 16)	46.24 (9.87)	45.00 (9.09)	MC: 0.208
Retinal hamartoma			
No (N = 40)	46.99 (10.39)	45.16 (8.63)	PC: 0.241
Yes (N = 9)	49.36 (6.89)	47.61 (3.81)	**MC: 0.023**
Diagnosis			
Childhood (N = 27)	47.2 (10.1)	43.46 (9.04)	PC: 0.519
Adult (N = 20)	48.04 (9.55)	48.25 (5.47)	**MC:0.023**

Bold highlights statistically significant results

TAND, TSC-associated neuropsychiatric disorders; AML, angiomyolipoma; LAM, lymphangioleiomyomatosis; SD, standard deviation; PC, physical component; MC, mental component

In patient subgroups that did not present clinical manifestations, the values of the physical and mental components fell within the range of the Spanish average, but decreased as the condition worsened (Table 6).

A statistically significant difference was observed in the mental health component between patients diagnosed in childhood and those diagnosed in adulthood (Table 6).

Finally, the influence of mTOR inhibitors on quality of life was analyzed, and no statistically significant differences in the mental or physical health components were found between those receiving treatment and those who were not.

## Discussion

Tuberous sclerosis is a complex disorder with multiple organ involvement and low prevalence, with such a broad spread of cases that it is difficult to conduct studies in this population. This article describes the largest Spanish cohort of adult patients with tuberous sclerosis, which is a little described clinical condition, given the tendency to describe cohorts of child patients. It also compares the clinical characteristics of patients diagnosed in childhood versus adulthood, and explores in greater depth the parameter of quality of life, which is an important variable when dealing with chronic conditions [23-25]

The data collected show that practically half the patients being followed in our series (45.6%) were diagnosed as adults; neurological involvement in the form of epilepsy was most frequently found in those diagnosed in childhood, while in adults, cortical dysplasia, cutaneous lesions and renal involvement predominated. Patients with onset of symptoms in adulthood present a mild form of the condition, with less intellectual disability and less frequency of epileptic seizures. This difference may be due to the genetic involvement [18, 26], with *TSC2* predominating among those diagnosed in childhood [27]. Also worthy of note is the presence of a significantly higher number of renal AMLs in patients with *TSC2* compared to *TSC1*, which coincides with the literature where the presence of *TSC2* is reported to be of greater severity. We found the same phenomenon in the use of mTOR inhibitors, in which only patients with *TSC2* were on mTOR inhibitors, whereas none of the patients with *TSC1* would have required them [24]. Those diagnosed in childhood are also in greater need of this treatment and have a worse quality of life.

Genetic tests were conducted on 77,2% of the patients. This figure is higher than that found in other studies, such as the TOSCA (43.1%) [28, 29], and may be due to the fact that our unit is multidisciplinary, with centralized patient assessment and uniform tests.

With respect to some of the typical clinical characteristics, we would like to highlight especially the differences found in epilepsy and cardiac rhabdomyoma; epilepsy was less prevalent in this study than has been reported elsewhere, 66.6% versus 75–90% [28, 30-32]. This could be related to the fact that this symptom is common in children, but less so in patients diagnosed in adulthood, which was 45.6% of our population. We found cardiac rhabdomyoma in 28% of patients in our cohort, a lower percentage than described in other series, which was expected, since this is a tumor that decreases naturally with age [33].

Treatment with mTOR inhibitors has been available for this condition for the last decade. The criteria for treatment were stipulated at the International TSC Consensus Conference of 2012, and refractory epilepsy was added later [8, 12, 13]. In comparison with other studies, the absolute number of patients in our cohort being treated with mTOR inhibitors was relatively small; nevertheless, 42.1% of patients in our series were receiving treatment with them, with a good safety profile.

A distinctive feature of our study is that it assesses the quality of life of adults with the condition across both physical and mental health components. As in other series, we found that quality of life was impaired in patients with TS, regardless of whether or not there was epilepsy or other cognitive disabilities [18, 23, 25].

When we compare the quality of life results of the patients in our cohort with the published quality of life results in the average Spanish population and in the stratification by over 60 years, we see that in the general health, vitality, social function and mental health, the quality of life is decreased in our cohort with respect to the average population with statistically significant differences. However, there are no differences with respect to those over 60 years of age, with the exception of the physical function scale where we found a higher mean in our cohort [34, 35].

The psychosocial aspect was the most affected, with a worse quality of life in those patients with a greater affectation, but there are significant differences in all domains of the SF-36 between patients with TS and the general population. This can also be seen when analyzing the patients according to the diagnosis in childhood or adulthood where we did not find statistically significant differences in the quality of life in the physical component, but in the mental component with *p* = 0.023; having a worse quality of life those diagnosed in childhood.

This may suggest the need for psychosocial counselling, both for patients and their families. Although in our study we have not analyzed the quality of life of the families is an important point to focus as well.

The limitations of this study are that patients come from a single center, the number of patients in the sample is small in absolute terms, and it is a retrospective study.

Is surprising the small number of patients mentioned as transition from childhood to adulthood. This is justified because before the multidisciplinary consultation was created, these patients made the transition to the specific adult specialist based on their clinical manifestations and were subsequently collected again when the multidisciplinary consultation was created, so part of the patients included in the multidisciplinary consultation reason, were also assessed from childhood.

Despite these limitations, we highlight as strengths that the number of patients is large for a condition that is relatively rare, and that it was conducted at a referral center with experience of the pathology. In addition, it contributes a novel view from the point of view of assessment of adults, treatment, and quality of life.

## Conclusions

TS patients diagnosed in adulthood have a milder form of disease presentation, with less need for treatment and less deterioration in quality of life. Treatment with mTOR inhibitors achieves stability of lesions in most patients, with a low percentage of serious adverse effects.

The quality of life of patients with TS is lower than that of the general Spanish population.

## Data Availability

The datasets used and analysed during the current study are available from the corresponding author on reasonable request.

## Abbreviations

TS: Tuberous sclerosis
TSC: Tuberous sclerosis complex
AML: Angiomyolipoma
LAM: Lymphangioleiomyomatosis
SEGA: Subependymal giant cell astrocytoma
TAND: TSC-associated neuropsychiatric disorders
mTOR: Mammalian target of rapamycin
SD: Standard deviation
MR: Magnetic resonance

## References

[1] Yates JR (2006). Tuberous sclerosis. Eur J Hum Genet.

[2] O'Callaghan FJ, Shiell AW, Osborne JP, Martyn CN (1998). Prevalence of tuberous sclerosis estimated by capture-recapture analysis. Lancet.

[3] Osborne JP, Fryer A, Webb D (1991). Epidemiology of tuberous sclerosis. Ann N Y Acad Sci.

[4] Ebrahimi-Fakhari D, Mann LL, Poryo M (2018). Incidence of tuberous sclerosis and age at first diagnosis: new data and emerging trends from a national, prospective surveillance study. Orphanet J Rare Dis.

[5] Ebrahimi-Fakhari D, Mann LL, Poryo M (2019). Correction to: Incidence of tuberous sclerosis and age at first diagnosis: new data and emerging trends from a national, prospective surveillance study. Orphanet J Rare Dis.

[6] Crino PB, Nathanson KL, Henske EP (2006). The tuberous sclerosis complex. N Engl J Med.

[7] Northrup H, Krueger DA; International Tuberous Sclerosis Complex Consensus Group. Tuberous sclerosis complex diagnostic criteria update: recommendations of the 2012 International tuberous sclerosis complex consensus conference. Pediatr Neurol. 2013;49(4):243–254. 10.1016/j.pediatrneurol.2013.08.001 10.1016/j.pediatrneurol.2013.08.001 PMC4080684 24053982

[8] Krueger DA, Northrup H; International Tuberous Sclerosis Complex Consensus Group. Tuberous sclerosis complex surveillance and management: recommendations of the 2012 International tuberous sclerosis complex consensus conference. Pediatr Neurol. 2013;49(4):255–265. 10.1016/j.pediatrneurol.2013.08.002 10.1016/j.pediatrneurol.2013.08.002 PMC4058297 24053983

[9] Franz DN, Belousova E, Sparagana S (2013). Efficacy and safety of everolimus for subependymal giant cell astrocytomas associated with tuberous sclerosis complex (EXIST-1): a multicentre, randomised, placebo-controlled phase 3 trial. Lancet.

[10] Fogarasi A, De Waele L, Bartalini G (2016). EFFECTS: an expanded access program of everolimus for patients with subependymal giant cell astrocytoma associated with tuberous sclerosis complex. BMC Neurol.

[11] Trelinska J, Dachowska I, Baranska D (2017). Maintenance therapy with everolimus for subependymal giant cell astrocytoma in patients with tuberous sclerosis (the EMINENTS study). Pediatr Blood Cancer.

[12] Krueger DA, Wilfong AA, Mays M (2016). Long-term treatment of epilepsy with everolimus in tuberous sclerosis. Neurology.

[13] French JA, Lawson JA, Yapici Z (2016). Adjunctive everolimus therapy for treatment-resistant focal-onset seizures associated with tuberous sclerosis (EXIST-3): a phase 3, randomised, double-blind, placebo-controlled study. Lancet.

[14] Bissler JJ, Kingswood JC, Radzikowska E (2013). Everolimus for angiomyolipoma associated with tuberous sclerosis complex or sporadic lymphangioleiomyomatosis (EXIST-2): a multicentre, randomised, double-blind, placebo-controlled trial. Lancet.

[15] Bissler JJ, Kingswood JC, Radzikowska E (2017). Everolimus long-term use in patients with tuberous sclerosis complex: four-year update of the EXIST-2 study. PLoS ONE.

[16] Marques R, Belousova E, Benedik MP (2019). Treatment patterns and use of resources in patients with tuberous sclerosis complex: insights from the TOSCA registry. Front Neurol.

[17] Auvin S, Bissler JJ, Cottin V (2019). A step-wise approach for establishing a multidisciplinary team for the management of tuberous sclerosis complex: a Delphi consensus report. Orphanet J Rare Dis.

[18] Pfirmann P, Aupy J, Jambon E (2020). Description of a multidisciplinary model of care in a French cohort of adult patients with tuberous sclerosis complex. J Med Genet.

[19] BautistaAlonso RE, ArtajonaRodrigo E, PovarEcheverría M (2019). Tuberous sclerosis complex in a third level hospital: need for integral management. Esclerosis tuberosa en un hospital de tercer nivel: necesidad de un abordaje integral. Med Clin Barc..

[20] Peron A, Canevini MP, Ghelma F, Di Marco F, Vignoli A (2018). Healthcare transition from childhood to adulthood in Tuberous Sclerosis Complex. Am J Med Genet C Semin Med Genet.

[21] Nellist M, Brouwer RW, Kockx CE (2015). Targeted next generation sequencing reveals previously unidentified TSC1 and TSC2 mutations. BMC Med Genet.

[22] Papadopoulou A, Dinopoulos A, Koutsodontis G (2018). Screening for TSC1 and TSC2 mutations using NGS in Greek children with tuberous sclerosis syndrome. Eur J Paediatr Neurol.

[23] Amin S, Mallick AA, Lux A, O'Callaghan F (2019). Quality of life in patients with Tuberous Sclerosis Complex (TSC). Eur J Paediatr Neurol.

[24] Rentz AM, Skalicky AM, Liu Z (2015). Tuberous sclerosis complex: a survey of health care resource use and health burden. Pediatr Neurol.

[25] Fong CY, Ng K, Kong AN (2019). Quality of life of children with tuberous sclerosis complex. Arch Dis Child.

[26] Verhoef S, Vrtel R, van Essen T (1995). Somatic mosaicism and clinical variation in tuberous sclerosis complex. Lancet.

[27] Dabora SL, Jozwiak S, Franz DN (2001). Mutational analysis in a cohort of 224 tuberous sclerosis patients indicates increased severity of TSC2, compared with TSC1, disease in multiple organs. Am J Hum Genet.

[28] Kingswood JC, d'Augères GB, Belousova E (2017). TuberOus SClerosis registry to increase disease awareness (TOSCA)-baseline data on 2093 patients. Orphanet J Rare Dis.

[29] Kingswood JC, Bruzzi P, Curatolo P (2014). TOSCA-first international registry to address knowledge gaps in the natural history and management of tuberous sclerosis complex. Orphanet J Rare Dis.

[30] Lu DS, Karas PJ, Krueger DA, Weiner HL (2018). Central nervous system manifestations of tuberous sclerosis complex. Am J Med Genet C Semin Med Genet.

[31] Cotter JA (2020). An update on the central nervous system manifestations of tuberous sclerosis complex. Acta Neuropathol.

[32] Curatolo P, Moavero R, de Vries PJ (2015). Neurological and neuropsychiatric aspects of tuberous sclerosis complex. Lancet Neurol.

[33] Hinton RB, Prakash A, Romp RL, Krueger DA, Knilans TK. International Tuberous Sclerosis Consensus Group. Cardiovascular manifestations of tuberous sclerosis complex and summary of the revised diagnostic criteria and surveillance and management recommendations from the International Tuberous Sclerosis Consensus Group. J Am Heart Assoc. 2014;3(6):e001493. 10.1161/JAHA.114.001493 10.1161/JAHA.114.001493 PMC4338742 25424575

[34] Vilagut G, Valderas JM, Ferrer M, Garin O, López-García E, Alonso J (2008). Interpretación de los cuestionarios de salud SF-36 y SF-12 en España: componentes físico y mental [Interpretation of SF-36 and SF-12 questionnaires in Spain: physical and mental components]. Med Clin (Barc).

[35] Alonso J, Regidor E, Barrio G, Prieto L, Rodríguez C, de la Fuente L (1998). Valores poblacionales de referencia de la versión española del Cuestionario de Salud SF-36 [Population reference values of the Spanish version of the Health Questionnaire SF-36]. Med Clin (Barc).

